# Multifarious Indigenous Diazotrophic Rhizobacteria of Rice (*Oryza sativa* L.) Rhizosphere and Their Effect on Plant Growth Promotion

**DOI:** 10.3389/fnut.2021.781764

**Published:** 2022-01-13

**Authors:** Mohammad Imran Mir, Bee Hameeda, Humera Quadriya, B. Kiran Kumar, Noshin Ilyas, Ali Tan Kee Zuan, Hesham Ali El Enshasy, Daniel Joe Dailin, Hazem S. Kassem, Abdul Gafur, R. Z. Sayyed

**Affiliations:** ^1^Department of Botany, University College of Science, Osmania University, Hyderabad, India; ^2^Department of Microbiology, University College of Science, Osmania University, Hyderabad, India; ^3^Department of Botany, Pir Mehr Ali Shah Arid Agriculture University, Rawalpindi, Pakistan; ^4^Department of Land Management, Faculty of Agriculture, Universiti Putra Malaysia, Serdang, Malaysia; ^5^Institute of Bioproduct Development, Universiti Teknologi Malaysia, Skudai, Malaysia; ^6^School of Chemical and Energy Engineering, Faculty of Engineering, Universiti Teknologi Malaysia, Skudai, Malaysia; ^7^City of Scientific Research and Technology Applications, New Burg Al Arab, Alexandria, Egypt; ^8^Department of Agricultural Extension and Rural Society, College of Food and Agriculture Sciences, King Saud University, Riyadh, Saudi Arabia; ^9^Sinarmas Forestry Corporate Research and Development, Perawang, Indonesia; ^10^Asian Plant Growth Promoting Rhizobacteria Society (PGPR) for Sustainable Agriculture, Auburn University, Auburn, AL, United States

**Keywords:** antagonism, biofilm formation, diazotrophic rhizobacteria, nutrition, plant growth promotion, rice rhizosphere

## Abstract

A diverse group of rhizobacteria persists in the rhizospheric soil, on the surface of roots, or in association with rice plants. These bacteria colonize plant root systems, enhance plant growth and crop yield. Indigenous rhizobacteria are known to promote soil health, grain production quality and serve as sustainable bioinoculant. The present study was aimed to isolate, identify and characterize indigenous plant growth promoting (PGP) diazotrophic bacteria associated with the rhizosphere of rice fields from different areas of Jammu and Kashmir, India. A total of 15 bacteria were isolated and evaluated for various PGP traits, antagonistic activity against phytopathogens, production of hydrolytic enzymes and biofilm formation under *in-vitro* conditions. The majority of the isolated bacteria were Gram-negative. Out of 15 bacterial isolates, nine isolates produced IAA (12.24 ± 2.86 to 250.3 ± 1.15 μg/ml), 6 isolates exhibited phosphate solubilization activity (36.69 ± 1.63 to 312.4 ± 1.15 μg/ml), 7 isolates exhibited rock phosphate solubilization while 5 isolates solubilized zinc (10–18 mm), 7 isolates showed siderophore production, 8 isolates exhibited HCN production, 6 isolates exhibited aminocyclopropane-1-carboxylate (ACC) deaminase activity, 13 isolates exhibited cellulase activity, nine isolates exhibited amylase and lipase activity and six isolates exhibited chitinase activity. In addition, 5 isolates showed amplification with *the nifH* gene and showed a significant amount of nitrogenase activity in a range of 0.127–4.39 μmol C_2_H_4_/mg protein/h. Five isolates viz., IHK-1, IHK-3, IHK-13, IHK-15 and IHK-25 exhibited most PGP attributes and successfully limited the mycelial growth of *Rhizoctonia solani* and *Fusarium* oxysporum *in-vitro*. All the five bacterial isolates were identified based on morphological, biochemical and 16S rDNA gene sequencing study, as *Stenotrophomonas maltophilia, Enterobacter* sp., *Bacillus* sp., *Ochrobactrum haematophilum* and *Pseudomonas aeruginosa*. Rice plants developed from seeds inoculated with these PGP strains individually had considerably higher germination percentage, seed vigor index and total dry biomass when compared to control. These findings strongly imply that the PGP diazotrophic bacteria identified in this work could be employed as plant growth stimulators in rice.

## Introduction

Rice (*Oryza sativa* L.) is the world's most significant food crop, providing a staple diet for almost 3 billion people, or roughly half of the world population. India tops in the cultivated area for rice while ranks next to China in Global rice production (1). With the present trend of rice consumption, by 2025, it would possibly be 4.6 billion people, and to meet the demand, there must be a 20% rise in its production. Nitrogen plays a crucial role in plant growth and development. Dinitrogen gas that occupies 80% of the atmosphere is not readily available to plants. The plant-available forms of nitrogen are ammonium and nitrate in the soil. Nitrogen is an important limiting factor on rice yields, and hence an increase in rice productivity will inevitably involve a higher demand for nitrogen-containing inorganic fertilizers (2). Farmers worldwide use excess chemical fertilizers (especially nitrogen-containing chemical fertilizers) and pesticides to boost rice production. World nitrogen demand increased from 105.148 to 111.591 thousand tons during 2016–2022 as per FAO 2022 report (World fertilizer trends and outlook to 2022 FAO-UN).

On the other hand, chemical fertilizers are not only expensive for farmers but can also pollute the environment by discharging nitrates and nitrogen oxides into the soil, groundwater and air. They also harm vital soil microbes, reduce soil fertility, promote pest resistance, remain in food grains and adversely affect human health (3, 4). Therefore, substitute techniques must be prioritized that improve soil fertility and enhance rice production without the use of chemical fertilizers.

Sustainable agricultural practices like employing plant growth-promoting rhizobacteria (PGPR) or microbial inoculants are coming to the limelight in intensive agriculture globally. The rhizosphere has long been the focus of agricultural research due to its importance in crop productivity, soil health and sustainable agriculture. The rhizosphere is a narrow zone of soil surrounding the root, which is under the immediate influence of the root system (5). This zone is nutrient-dense compared to the bulk soil because of the concentration of various organic compounds released by roots through exudation, secretion, and deposition. Because microbes can exploit these organic molecules as carbon and energy sources, microbial growth and activity are pretty severe in the rhizosphere. As a result, 10–100 times higher microbial concentration in rhizospheric soils than bulk soils is observed (6–8). Rhizobacteria are plant-associated bacteria that can colonize roots and are categorized into three types based on their influence on plant growth: beneficial, harmful, and neutral. Beneficial rhizobacteria that promote plant growth are usually called a plant- growth-promoting rhizobacteria or PGPR (9). Diazotrophic bacteria, which can convert N_2_ into ammonia for plants, are also members of the PGPR (10). PGPR improves plant growth through a variety of mechanisms, including direct and indirect mechanisms. Direct mechanisms of plant growth by PGPR include the capacity to synthesize plant growth hormones such as auxins, gibberellins and cytokinins (11, 12), enhancing symbiotic nitrogen fixation (13), solubilizing minerals (e.g., phosphorus, zinc, potassium, etc.) (14, 15), siderophore production (iron chelators) (16, 17) and production of ACC deaminase enzyme which helps in lowering ethylene levels in developing plant roots, resulting in increased root growth (18, 19). The biocontrol capabilities of PGPR in response to biotic stress by generating antibiotics and cyanide, as well as fungal cell wall lysing enzymes that limit the growth of soil-borne phytopathogens, are the main indirect mechanisms (13, 20). Many bacteria from the rhizosphere soil have been isolated and identified in recent decades, including *Azospirillum, Klebsiella, Pseudomonas, Azotobacter, Alcaligens, Enterobacter, Burkholderia, Arthobacter, Serratia, Bacillus* and *Stenotrophomonas* have reported (17, 21–24). PGPR is increasingly being used in agriculture as soil nutrient supplements and bio-control agents as they are ecofriendly alternative to chemicals used. The present study aims to isolate, characterize and identify efficient PGP diazotrophic bacteria from rice rhizosphere.

## Materials and Methods

### Soil Sample Collection, Media Used and Isolation of Diazotrophs

**Figure 1:**
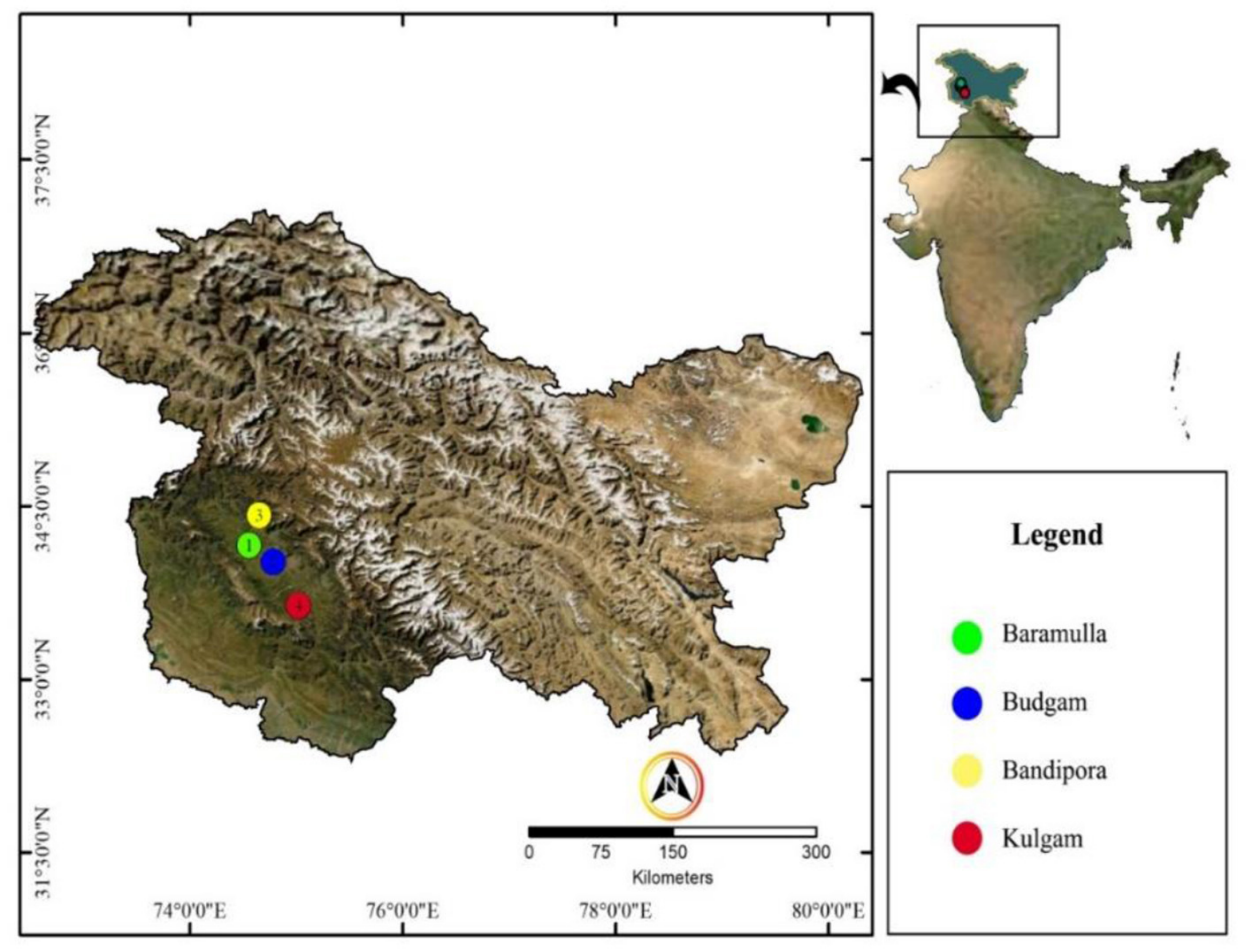
Map showing soil sample collection sites.

Rhizosphere soil of rice was collected from different agricultural field sites of Jammu & Kashmir, India. The place of collection and the isolate number are represented in Figure 1 and Table 1.

**Table 1:** Collection details of samples.

**Bacterial isolates labeled**	**Source of isolation**	**Place of collection**	**Coordinates**
IHK-1, IHK-2, IHK-3, IHK-4	Rice rhizosphere	Baramulla, J & K	34°12′7.7328″N 74°20′53.7324″E
IHK-5, IHK-6, IHK-7, IHK-8	Rice rhizosphere	Budgam, J & K, India.	34°1′12″N 74°46′48″E
IHK-9, IHK-10, IHK-11, IHK-12	Rice rhizosphere	Bandipora, J & K	34°25′12″N 74°39′00″E
IHK-13, IHK-15, IHK-25	Rice rhizosphere	Kulgam, J & K	33°38′24″N 75°01′12″E

Rice plants were uprooted from a depth of 0–5 cm for rhizosphere soil sampling. The samples were then immediately packed in sterile polythene bags, carefully transported to the laboratory, and stored at a temperature of 4°C. For isolation of bacteria from rice rhizosphere, serial dilution spread-plate technique was employed. 10 g of the rhizospheric soil sample was placed in 250 ml of Erlenmeyer flask containing 90 ml of sterile saline solution (0.85% w/v NaCl) and agitated (150 rpm) for 20 min at 30°C. Serial dilution was made, and 0.1 ml aliquots (10^−3^-10^−5^) were spread on plates containing combined carbon/rennie medium, a medium free of nitrogen sources. The Rennie medium was composed of two solutions with the following ingredients: Solution A consists of KH_2_P0_4_ 0.2 g; K_2_HPO_4_ 0.8 g; NaCl 0. I g; Na_2_MoO_4_.2H_2_O 25.0 mg; Na_2_FeEDTA 28.0 mg; yeast extract 100 mg; sucrose 5 g; mannitol 5 g; sodium lactate 0.5 mL (60%, v/v); N- free agar 20 g; distilled water 900 mL. Solution B consists of CaCl_2_ 0.06 g; MgS0_4_.7H_2_0 0.2 g; distilled water 100 mL. Both solutions were autoclaved separately, cooled and combined; filter-sterilized para-aminobenzoic acid (10 μg/l) and biotin (5 μg/l) were added and the pH of the medium was set to 7.0 (25, 26). The plates were incubated for 3–4 days at 30°C. After incubation, morphologically different colonies appearing on the medium were picked out using a sterile inoculating loop and were further purified by the streak plate method. The purified cultures were stored in nutrient agar slants at 4°C and 30% glycerol stocks at −80°C for further studies.

### Screening of Isolates for Multiple Plant Growth-Promoting Traits

#### Screening of Nitrogen-Fixing Activity—Growth on NFB Medium, Screening of NifH Genes and ARA Assay

The ability of the isolated bacteria to fix nitrogen was evaluated by growing them on an nitrogen-free solid malate (NFM) medium as described by Döbereiner et al. (27). The medium contains malic acid 0.5 g; KH_2_PO_4_ 0.5 g; MgSO_4_.7H_2_O 0.2 g; NaCl 0.1g; CaCl_2_ 0.02 g; Na_2_MoO_4_ 0.002 g; MnSO_4_.H_2_O 0.01 g; EDTA 1.64% - 4 ml; bromothymol blue 0.5% −3 ml; biotin 0.1 mg; KOH 4.5 g; distilled water 1,000 ml and the pH was adjusted to 6.8. The isolated diazotrophic bacteria were streaked on NFB medium, incubated at 30°C for 48–72 h. The bacterial isolates that changed the color of the media to blue were assumed as N_2_ fixers.

The N_2_-fixing capability of the isolated bacterial was further screened by the presence of *nifH* genes responsible for nitrogen fixation. For this, genomic DNA was extracted from the pure cultures of the isolated bacteria using GSure bacterial genomic DNA isolation kit (GCC Biotech) following the manufacturer's instructions. Genomic DNA of the isolated diazotrophic bacteria was used as template in PCR for the amplification of *nifH* gene using specific primers: polR (5′-ATS GCC ATC ATY TCR CCG GA−3′) and polF (5′-TGC GAY CCS AAR GCB GAC TC-3′) as described by Poly et al. (28). The amplification reaction was performed in a total volume of 25 μl. The reaction mixture contained: 2 μl 10x PCR buffer, 1.5 μl of 2 mM each of deoxynucleoside triphosphate (dATP, dCTP, dTTP and dGTP), 1 μl of each forward PolF and reverse PolR primer, 3 μl of template DNA (100 ng) and 0.4 μl of (2 U/l) Taq polymerase; the final volume was made into 25 μl using NFW (nuclease-free water). The thermocycling condition consisted of an initial denaturation step at 94°C for 5 min, followed by 35 cycles of denaturation at 94°C for 1 min, an annealing temperature of 55°C for 1 min, extension at 72°C for 2 min and one cycle at 72°C for 10 min for final elongation (28) (Eppendorf Mastercycler Nexus). Amplification products were analyzed by 1.5% agarose gel electrophoresis stained with ethidium bromide, visualized by gel document system (BIO RAD) and the band size calculated by comparison with the 100-bp ladder length standard.

The nitrogen-fixing ability of the isolated bacteria was further evaluated by ARA assay, as per the methodology of Hardy et al. (29). Isolates were inoculated into semi-solid nitrogen-free medium vials and were grown at 30°C for 72 h. The vials were covered with suba seals and 10% (v/v) of the air phase was substituted with acetylene and incubated for 1 h at 30°C. After an incubation period, 1 ml of the above sample was withdrawn using a sterile disposable microsyringe and injected into a GC (Agilent 7890B), equipped with a flame ionization detector (FID) to detect ethylene (C_2_H_4_) and acetylene gas. Based on the standard curve and peak-area percentage, the rate of N_2_ fixation was calculated as the amount of ethylene accumulated (μmol C_2_H_4_/ mg protein/hour).

#### Qualitative and Quantitative Estimation of IAA Production

Qualitative estimation of IAA production by bacterial isolates was performed as per the method described by Williams and Signer (30). In brief, bacterial isolates were grown on NA medium supplemented with 5 mM DL—tryptophan at 30°C for 72 h. After incubation, the inoculated spots were overlaid with nitrocellulose membrane (NCM) disk that had been pre-saturated with Salkowski reagent and observed for the appearance of the pink color of NCM disks. Bacterial isolates which were positive in plate culture conditions were tested for quantitative assessment of IAA in broth culture by colorimetry as reported by Gordon and Weber (31).

#### Phosphate and Rock Phosphate Solubilization

The ability to solubilize phosphate was tested by spot inoculating bacterial isolates on the National Botanical Research Institute's Phosphate Growth (NBRIP) medium containing tricalcium phosphate (TCP) as insoluble form of phosphate. The formation of halo zones around the colonies was considered positive for phosphate solubilization. For quantitative phosphate estimation, the log phase culture of isolates was grown in NBRIP broth at 30°C for 7 days. Following the centrifugation at 10,000 rpm, for 10 min, the supernatant was assayed for P solubilization (32).

The ability to solubilize rock phosphate was carried out by spot inoculating isolated diazotrophic bacteria on TRP (Tris-buffered rock phosphate) agar medium amended with methyl red pH indicator and 100 mM glucose as sole carbon source. The development of red coloration around the bacterial colonies on the TRP agar medium indicated rock phosphate (RP) solubilization (33).

#### Zinc Solubilizing Activity

The ability of the isolated diazotrophic bacteria to solubilize zinc was tested by growing them on tris mineral salt agar medium at 30°C for 72 h. After incubation, the diameter of the clearing zones around the colonies was measured (34, 35).

#### Siderophore Production

Siderophore production was carried out by growing the isolates on Chrome Azurol S (CAS) agar at 30°C for 2–3 days in the dark and observing the development of orange halo zones around the bacterial colonies an indication of siderophore production (36).

#### Hydrocyanic Acid (HCN) Production

Qualitative estimation of HCN of the isolated diazotrophic bacteria was carried out using Bakker and Schippers method (37). Briefly, all the isolates were streaked on a nutrient agar plate supplemented with 4.4 % glycine. A Whatman filter paper No. 1 soaked in a solution of 2% sodium carbonate in 0.5% picric acid solution was fitted to the underside of the plate lids. The plates were sealed with parafilm and incubated at 28 ± 2°C for 5 days. Change in color of filter paper from yellow to brown, medium brown (++), or strong brown (+++) indicates the production of hydrocyanic acid.

#### Detection of Ammonia Production

The bacterial isolates were tested for ammonia production in peptone water as per the methodology of Di-Benedetto et al. (4). In brief, bacterial cultures were inoculated in test tubes containing peptone water broth (10 mL) and incubated for 3 days at 30°C. After the incubation period, 0.5 mL of Nessler's reagent was added to each tube, development of yellow to the brown color indicated ammonia production.

#### Screening for ACC Deaminase Activity

The qualitative estimation of ACC (1-aminocyclopropane-1-carboxylate**)** deaminase was carried out as per the methodology prescribed by Penrose and Glick (38). Bacterial isolates were grown in tryptic soy broth for 48 h at 150 rpm at 30°C. Cell pellet collected by centrifugation at 8,000 rpm for 5 min was washed three times with sterile 0.1 M Tris-HCl (pH 7.5) and spot inoculated on plates containing DF (Dworkin and Foster) salt minimal medium supplemented with 3 mM ACC. DF medium without ACC served as the negative control, while DF medium containing (NH_4_)_2_SO_4_ (0.2% w/v) served as the positive control. The plates were incubated at 30°C for 4–5 days. The ability of the isolates to grow on ACC plates indicated that they exhibited ACC deaminase activity.

#### Screening of Isolates for Hydrolytic Enzymes- Chitinase, Lipase, Protease, Amylase and Cellulase

Qualitative assay for protease and lipase production was performed by spot inoculating actively grown cultures on sterile skim milk agar and Tween 80 agar media plates, incubated for 48–72 h at 30°C. Halo zone formation around the bacterial colonies indicated protease and lipase production (22).

Amylase production by isolated diazotrophic bacteria was carried out by spot inoculating the actively grown cultures on a starch agar medium and incubated for 48–72 h at 28 ± 2°C. After incubation, the plates were saturated with iodine solution, held for a min, and then drained off. Appearance of colorless zones around colonies suggest amylase production (39).

Production of cellulase by isolated diazotrophic bacteria was carried out as per the method of Hendricks et al. (40). In brief, 5 μl of actively grown bacterial cultures were spot inoculated on carboxymethylcellulose congo red media with the following composition (carboxymethylcellulose 0.5 g; K_2_HPO_4_ 0.099 g; MgSO_4_.7H_2_O 0.049 g; yeast extract 0.05 g; congo red 0.05 g; agar 20 g; distilled water 1 L; pH 7.2). Plates were incubated at 28 ± 2°C for 72 h, halo zone formation implies cellulase production.

Qualitative assay for chitinase activity was performed by spot inoculating the actively growing rhizobacterial strains on the colloidal chitin incorporated medium (chitin minimal medium) and incubated at 28 ± 2°C for 3–4 days. The formation of halo zones around the colonies implies chitinase production (41).

#### Assessment of Antifungal Activity

*Rhizoctonia solani* and *Fusarium oxysporum* were procured from International Crops Research Institute for the Semi-Arid Tropics, Patancheru, Hyderabad, India. A 5 mm mycelial mat of each soil-borne fungus was cut and placed in the middle of the Petri plate containing potato dextrose agar (PDA). Actively grown cultures of each bacterial isolates were streaked in a straight line on one side of a 90 mm diameter Petri dish, with a space of 2 cm between the fungus and the test culture and the plates were incubated at 28 ± 2°C for 7 days. Control plates had only fungus. For each isolate, three independent replications were maintained. Inhibition of fungal growth was measured after 7 days and the percent inhibition over control was calculated employing the following formula:


$$\begin{array}{l} \text{I}=\frac{\text{C}-\text{T}}{\text{C}}x\;100 \end{array}$$

Where I = inhibition % of mycelial growth, C = radial growth of the pathogen without antagonists, T = radial growth of the pathogen with antagonists.

#### *In-vitro* Biofilm Formation

All the bacterial isolates were cultured in polypropylene tubes containing 10 ml of nutrient broth, incubated for 10 days at 30°C. The medium was decanted after the incubation period and the tubes were rinsed with phosphate-buffered saline of pH 7.3 and dried, then 1% of crystal violet stain was added and the tubes were incubated for 45 min at room temperature. The excess crystal violet was decanted from tubes. The tubes were checked for a purple ring at the top, which indicates the development of a biofilm (42).

### Identification of the Diazotrophic Bacteria

#### Phenotypic Characterization

Bacterial isolates were identified based on colony morphology, Gram staining and biochemical tests according to Bergey's Manual of Systematic Bacteriology (1994).

#### Molecular Characterization

Genomic DNA was extracted using GSure bacterial genomic DNA isolation kit (GCC Biotech). For 16S rRNA gene amplification, specific bacteria primers FGPS6 (5′ GGA GAG TTA GAT CTT GGC TCA G 3′) as forward and FGPS1509 (5′ AAG GAG GGG ATC CAG CCG CA 3′) as reverse were used according to Normand et al. (43). The PCR reaction setup and thermal profiling conditions were performed according to methods described by Zakhia et al. (44). Amplified PCR products of 16S ribosomal gene were confirmed by electrophoresis in 1% agarose gels containing ethidium bromide and visualized using UV-transilluminator. The 16S rDNA gene sequences of the bacterial isolates obtained were matched with available gene sequences using BLAST and aligned employing the Clustal -W program. Phylogenetic trees were constructed using the Neighbor-Joining method (45) and molecular evolutionary analyses were conducted using the MEGA X software (46). The nucleotide sequences of the bacterial strains were submitted to NCBI GenBank.

#### Evaluation of Plant Growth Promotion in Rice Treated With Potential Diazotrophic PGPR

Selected five potential diazotrophic plant growth-promoting bacteria (IHK-1, IHK-3, IHK-13, IHK-15, and IHK-25) were evaluated for their impact on rice seed germination and seedling vigor using the paper (roll) towel method. Seeds of rice cultivar BPT-5,204 were used in this study and the seeds were procured from Indian Institute of Rice Research (ICAR-IIRR) Rajendranagar, Hyderabad Telangana, India. Seeds were first sorted to eliminate broken, small seeds and then they were surface sterilized with 95% ethanol for 1 min followed by rinsing with 0.2% HgCl_2_ solution for 3 min and several washings with sterile distilled water (19). Bacterial isolates (IHK-1, IHK-3, IHK-13, IHK-15 and IHK-25) were grown in 50 mL nutrient broth with 100 μL inoculum size in a 150 mL Erlenmeyer flask and the flasks were kept on an incubator shaker (120 rpm), for 48 h at 28 ± 2°C. After incubation, the cultures were centrifuged at 8,000 rpm at 4°C for 10 min, to obtain the cell pellet. The obtained cell pellet was dissolved in sterile saline (0.8%) and the final population of preparation was adjusted to 10^8^ CFU/mL. The surface-disinfected rice seeds were dipped into respective cell pellet suspension amended with 1% sterile carboxymethylcellulose (CMC) for an hour. Bacterized seeds were placed in each germination paper and were incubated in a greenhouse for 15 days. The temperature was maintained and ranged between 22 and 28°C (average 26°C).

Three replications of the five treatments, i.e., T1-IHK-1, T2-IHK3, T3- IHK13, T4-IHK-15 and T5-IHK-25 were maintained. Seeds soaked in sterile distilled water served as control. After 15 days, seedlings were taken out of the germination papers, root and shoot growth, seed vigor index (SVI) and dry mass was recorded and compared with control (untreated). According to the International Seed Testing Association, the germination test was performed in a paper (roll) towel (ISTA, 1993). The following formula was used to determine the seed vigor index:


SVI=% germination x (mean shoot length+mean root length)

### Statistical Analysis

All the experiments were performed in triplicates and the data was statistically analyzed by R software Version 4.1.2 (https://cran.r-project.org/bin/windows/base/R-4.1.2.-win.exe). The comparison between treatment means was made using the Duncan's multiple range test (DMRT) at *p* < 0.05. Means with the same letter do not differ significantly at *p* < 0.05.

## Results

### Isolation of Rhizobacteria

A total of 15 rhizobacteria were secluded from the rice rhizosphere on rennie medium, a medium free of a nitrogen source. The isolates were labeled as IHK-1, IHK-2, IHK-3, IHK-4, IHK-5, IHK-6, IHK-7, IHK-8, IHK-9, IHK-10, IHK-11, IHK-12, IHK-13, IHK-15 and IHK-25 (Table 1).

### Detection of PGPR Traits of Diazotrophic Bacteria

The isolated bacteria were screened for multiple PGP activities, which are summarized in Table 2. The present findings revealed that not all 15 isolates had the same level of PGP activity. Varying levels of PGPR properties were found in the isolated bacteria. All the 15 bacterial isolates were examined qualitatively for nitrogen fixation ability in N-free solid malate medium (NFB medium) containing bromothymol blue as an indicator. All the bacterial isolates changed the color of the NFB medium from pale green to blue (Supplementary Figure S1A), suggested that the bacterial isolates can fix atmospheric nitrogen. Out of 15 bacterial isolates, five isolates (IHK-1, IHK-3, IHK-13, IHK-15 and IHK-25) showed amplification with *nifH* gene and the product of predicted size (360-bp) was obtained, indicating the presence of nitrogen-fixing genes in these diazotrophic bacteria (Supplementary Figure S1B). Acetylene reduction assay (ARA) showed eight isolated bacteria with nitrogenase activity ranging from 0.127 to 4.39 μmol C_2_H_4_/mg protein/hour. The highest ARA was observed in *Stenotrophomonas maltophilia* IHK-1, followed by *Bacillus sp*. IHK-13 (Table 2).

**Table 2:** PGP traits of the bacteria isolated from rice rhizosphere.

**Isolates**	**IAA (μg/ml)**	**PS (μg/ml)**	**SID**	**RPS**	**HCN**	**AMM**	**ACC**	**ZS zone (mm)**	**ARA (μmol C_2_H_4_ mg^−1^ protein h^−1^)**
IHK-1	161.6 ± 1.20	91.1 ± 0.88 (5.9)	++	+	++	+++	+	11	4.39
IHK-2	–	–	+	–	–	+	–	–	
IHK-3	230.6 ± 2.33	298.7 ± 1.25 (3.7)	+++	+	+++	+++	+	16	0.313
IHK-4	14.84 ± 1.62	–	–	–	–	+	–	–	0.127
IHK-5	–	–		–	+	++	–	–	
IHK-6	12.24 ± 2.86	–	–	–	–	+	–	–	
IHK-7	–	36.69 ± 1.63 (6.1)		+	–	+	–		0.195
IHK-8	–	–	–	–	+	+	–	–	0.174
IHK-9	21.03 ± 0.56	–	+	–	–	+	–	–	
IHK-10	–	–	–	–	–	+	+		
IHK-11	–	–	–	+	+	+	–		
IHK-12	16.84 ± 1.62	–		–	–	++	–	–	
IHK-13	101.3 ± 1.20	221.7 ± 0.40 (4.0)	++	+	+++	+++	+	10	3.21
IHK-15	160.3 ± 0.66	294.2 ± 0.97 (3.8)	+++	+	++	+++	+	14	0.29
IHK-25	250.3 ± 1.15	312.4 ± 1.15 (3.5)	+++	+	+++	+++	+	18	1.96

+++ *, strong activity*;

++ *, average activity*;

+ *, mild activity*;

– *, no activity; IAA, indole acetic acid; PS, phosphate solubilization; SID, siderophore production; RPS, rock phosphate solubilization; HCN, hydrocyanic acid production; AMM, ammonia production; ACC, 1-aminocyclopropane- 1 – carboxylate deaminase; ZS, zinc solubilization; ARA, acetylene reduction assay; numerical values are mean ± Standard Error (SE); values in the parentheses indicate the pH decreased from initial 7.0*.

All the bacterial isolates were tested qualitatively for indole acetic acid production. Nine bacterial isolates showed a substantial change in the color of the NCM disk to pink when sprayed with Salkowski's reagent confirms the presence of IAA (Supplementary Figure S2A) and were further selected for quantitative estimation of IAA in nutrient broth supplemented with tryptophan by spectrophotometric method. Production of IAA from these isolates ranged from 12.24 ± 2.86 to 250.3 ± 1.15 μg/ml. The highest production was observed by IHK-25 (250.3 ± 1.15 μg/ml), followed by isolates IHK-3, IHK-1, IHK-15 and IHK-13, which showed 230.6 ± 2.33 μg/ml, 161.6 ± 1.20 μg/ml, 160.3 ± 0.66 μg/ml and 101.3 ± 1.20 μg/ml, respectively. Bacterial isolates IHK-6, IHK-4, IHK-12 and IHK-9, showed the least production of 12.24 ± 2.86 μg/ml, 14.84 ± 1.62 μg/ml, 16.84 ± 1.62 μg/ml and 21.03 ± 0.56 μg/ml, respectively (Table 2).

Out of 15 bacterial isolates tested for phosphate solubilization, 6 isolates produced cleared zones around their respective colonies on NBRIP medium (Supplementary Figure S2B) and were selected for further quantification of phosphate solubilization studies. Maximum soluble phosphate of 312.4 ± 1.15 μg/ml was obtained with the supernatant of bacterial isolate IHK-25, followed by IHK-3 (298.7 ± 1.25 μg/ml), IHK-15 (294.2 ± 0.97 μg/ml), IHK-13 (221.7 ± 0.40) and IHK-1 (91.1 ± 0.88 μg/ml). Bacterial isolate IHK-7 showed the least soluble phosphate of 36.69 ± 1.63 μg/ml. The pH of the medium also showed a decrease from 7.0 in most of the isolates. In bacterial isolates IHK-25, IHK-3, IHK-15, the pH decreased from an initial 7.0 to a minimum of 3.5, 3.7, and 3.8, respectively (Table 2).

Production of siderophores by the bacterial isolates was analyzed by plating onto chrome azural S (CAS) agar medium. Of the 15 isolates, 7 isolates grew and showed orange/yellow halo around their respective colonies after three days of incubation at 30°C (Supplementary Figure S2C). Further, out of seven bacterial isolates, the IHK-3, IHK-15 and IHK-25 showed strong (+++) siderophore production, isolates IHK-1 and IHK-13 showed moderate (++), and mild activity (+) for siderophore production was shown by the remaining two isolates (IHK-2 and IHK-9).

Tris buffered rock phosphate (TRP) agar medium with a methyl red pH indicator was used for qualitative test for rock phosphate solubilization. Seven of the 15 bacterial isolates were identified to solubilize rock phosphate, as evidenced by red coloration around their respective colonies (Supplementary Figure S2D).

Eight of the 15 bacterial isolates tested positive for hydrogen cyanide generation, which was validated by the browning of the filter paper. Further, among eight bacterial isolates, it was observed that IHK-3, IHK-13 and IHK-25 showed the highest hydrocyanic acid production as they exhibited deep brown color. Bacterial isolates IHK-1 and IHK-15 showed moderate HCN production as they exhibited medium brown color, whereas the lowest (+) production was recorded in three isolates, namely IHK-5, IHK-8 and IHK-11, as they exhibited light brown color. The absence of brown color in filter paper discs indicated the inability of the isolates to produce HCN (Supplementary Figure S2E).

All 15 bacterial isolates showed ammonia production. Isolates were categorized based on color intensity into three groups viz, weak, moderate and strong ammonia producers. Among all the isolates screened, 5 isolates (IHK-1, IHK-3, IHK-13, IHK-15 and IHK-25) were strong ammonia producers, as evidenced by the change of color from yellow to deep brown on the addition of Nessler's reagent to 72 h old cultures grown in peptone water at 30 ± 2°C. 2 isolates exhibited moderate activity (++) and 8 isolates showed mild activity (+) for ammonia production (Supplementary Figure S2F; Table 2).

All the 15 bacterial isolates showed growth on DF minimal medium supplemented with ammonium sulfate, and only six isolates (IHK-1, IHK-3, IHK-10, IHK-13, IHK-15 and IHK-25) showed growth on DF minimal medium supplemented with ACC, indicated that bacterial isolates can produce ACC deaminase (Supplementary Figure S2G).

Among the fifteen bacterial isolates, five showed a zone of solubilization around the bacterial colonies on tris mineral salt medium amended with zinc carbonate as an insoluble form of zinc. The bacterial isolate IHK-25 showed a maximum solubilization zone of (18 mm), followed by IHK-3 (16 mm), IHK-15 (14 mm), IHK-1 (11mm), IHK-13 (10 mm) (Supplementary Figure S2H).

### Production of Hydrolyzing Extracellular Enzymes

PGPR produces several lytic enzymes in the rhizosphere responsible for the destruction of various components of fungal pathogens. The appearance of a clear zone surrounding the colonies on skim milk agar media was found as a sign of proteolytic enzyme production. Eleven of the 15 bacterial isolates tested positive for protease activity (Supplementary Figure S3A). Furthermore, out of eleven isolates, isolates IHK-1, IHK-3, IHK-13, IHK-15 and IHK-25 showed strong (+++) protease production.

Developing a halo zone around the colonies on carboxymethylcellulose congo-red media confirmed cellulase enzyme production by bacterial isolates. 13 bacterial isolates were tested positive for cellulase enzyme production (Supplementary Figure S3B). Furthermore, out of thirteen bacterial isolates, isolates IHK-1, IHK-3, IHK-13, IHK-15 and IHK-25 showed robust (+++) cellulase production.

Out of 15 bacterial isolates, nine isolates exhibited amylase activity (Supplementary Figure S3C), nine isolates exhibited lipase activity (Supplementary Figure S3D) and six isolates exhibited chitinase activity. Furthermore, out of 15 bacterial isolates, 5 isolates (IHK-1, IHK-3, IHK-13, IHK-15 and IHK-25) produced all five enzymes. The findings of the lytic enzyme production by the isolated bacteria are summarized in Table 3.

**Table 3:** Hydrolytic enzymes produced by bacteria isolated from the rhizosphere of rice.

**Isolates**	**Protease**	**Amylase**	**Chitinase**	**Cellulase**	**Lipase**	**No. of enzymes produced**
IHK-1	+++	+++	++	+++	++	5
IHK-2	+	–	–	++	+	3
IHK-3	+++	+++	++	+++	+++	5
IHK-4	++	–	+	+	++	4
IHK-5	–	+	–	–	–	1
IHK-6	+	–	–	+	–	2
IHK-7	–	–	–	+	–	1
IHK-8	++	++	–	–	–	2
IHK-9	–	+	–	++	+	3
IHK-10	+	–	–	+	–	2
IHK-11	+	–	–	++	–	2
IHK-12	–	+	–	++	++	3
IHK-13	+++	+++	+++	+++	++	5
IHK-15	+++	++	+	+++	++	5
IHK-25	+++	++	+	+++	+++	5

+++ *, strong activity*;

++ *, average activity*;

+ *, slight activity*.

### *In-vitro* Antagonistic Effect of Diazotrophic Bacteria Against Phytopathogens

The antagonistic activity of all 15 diazotrophic bacterial isolates against two soil-borne phytopathogens (*Rhizoctonia solani* and *Fusarium oxysporum*) was evaluated using the dual culture technique. The formation of inhibitory zones between the bacterium and fungal isolates confirmed the antagonistic effects. It has been found that mycelium growth of *Rhizoctonia solani* was inhibited by seven isolates, with the maximum suppression shown by isolate IHK-25 (78%), followed by IHK-13 (75%), IHK-3 (65%), IHK-1 (60%), IHK-15 (58%), IHK-4 (52%) and IHK-2 (51%) (Supplementary Figure S4A). Four isolates demonstrated >50% of suppression against *Fusarium oxysporum*, with isolate IHK-25 showing the highest level of inhibition of 80%, followed by IHK-13 (72%), IHK-3 (61%) and IHK-1 (54%) (Supplementary Figure S4B). Out of 15 bacterial isolates, four isolates viz., IHK-1, IHK-3, IHK-13 and IHK-25 exhibited inhibition potential against both phytopathogens tested (Table 4).

**Table 4:** Antagonistic activity against soil-borne phytopathogenic fungi.

**Isolates**	**% inhibition of phytopathogenic fungi**	
	**Rhizoctonia solani**	**Fusarium oxysporum**
IHK-1	60 ± 0.15^cd^	54 ± 0.32^d^
IHK-2	51 ± 0.26^d^	0
IHK-3	65 ± 0.14^c^	61 ± 0.1^c^
IHK-4	52 ± 0.34^d^	0
IHK-5	0	0
IHK-6	0	0
IHK-7	0	0
IHK-8	0	0
IHK-9	0	0
IHK-10	0	0
IHK-11	0	0
IHK-12	0	0
IHK-13	75 ± 0.18^ab^	72 ± 0.2^b^
IHK-15	58 ± 0.2^cd^	0
IHK-25	78 ± 0.32^a^	80 ± 0.3^a^

*Numerical values are mean ± SE; mean values in each column with the same letter do not differ significantly at p < 0.05. Comparison between treatment mean values was made using Duncan's multiple range test (DMRT). Mean values in each column with the same letters or symbol, do not differ significantly by DMRT (P = 0.05). Means values in each column with different letters differ significantly by DMRT (P = 0.05)*.

### Biofilm Formation

The formation of biofilms is an indirect way of measuring the rhizosphere colonizing activity of plant growth-promoting bacteria. All the bacterial isolates were characterized for their capability to form biofilms under *in-vitro* conditions. Among 15 bacterial isolates studied, 5 isolates (IHK-2, IHK-4, IHK-9, IHK-10 and IHK-11) formed very thin film, 2 isolates (IHK-6 and IHK-8) formed medium film, 5 isolates (IHK-1, IHK-3, IHK-13, IHK-15 and IHK-25) formed very thick biofilm whereas, three isolates (IHK-5, IHK-7 and IHK-12) did not form biofilms (Supplementary Figure S5).

### Identification of Isolates

#### Phenotypic Characterization

Based on the phenotypic characteristics, the isolated rhizobacteria strains were matched with those of the standard species using Bergey's Manual of Determinative Bacteriology and were initially identified as genus *Pseudomonas, Enterobacter*, Bacillus, *Stenotrophomonas* and *Ochrobactrum*. Morphological and biochemical characteristics of the isolated bacteria are represented in Supplementary Tables 1, 2 and Supplementary Figure S6).

#### Molecular Identification of the Selected Diazotrophic PGP Bacterial Isolates

**Figure 2:**
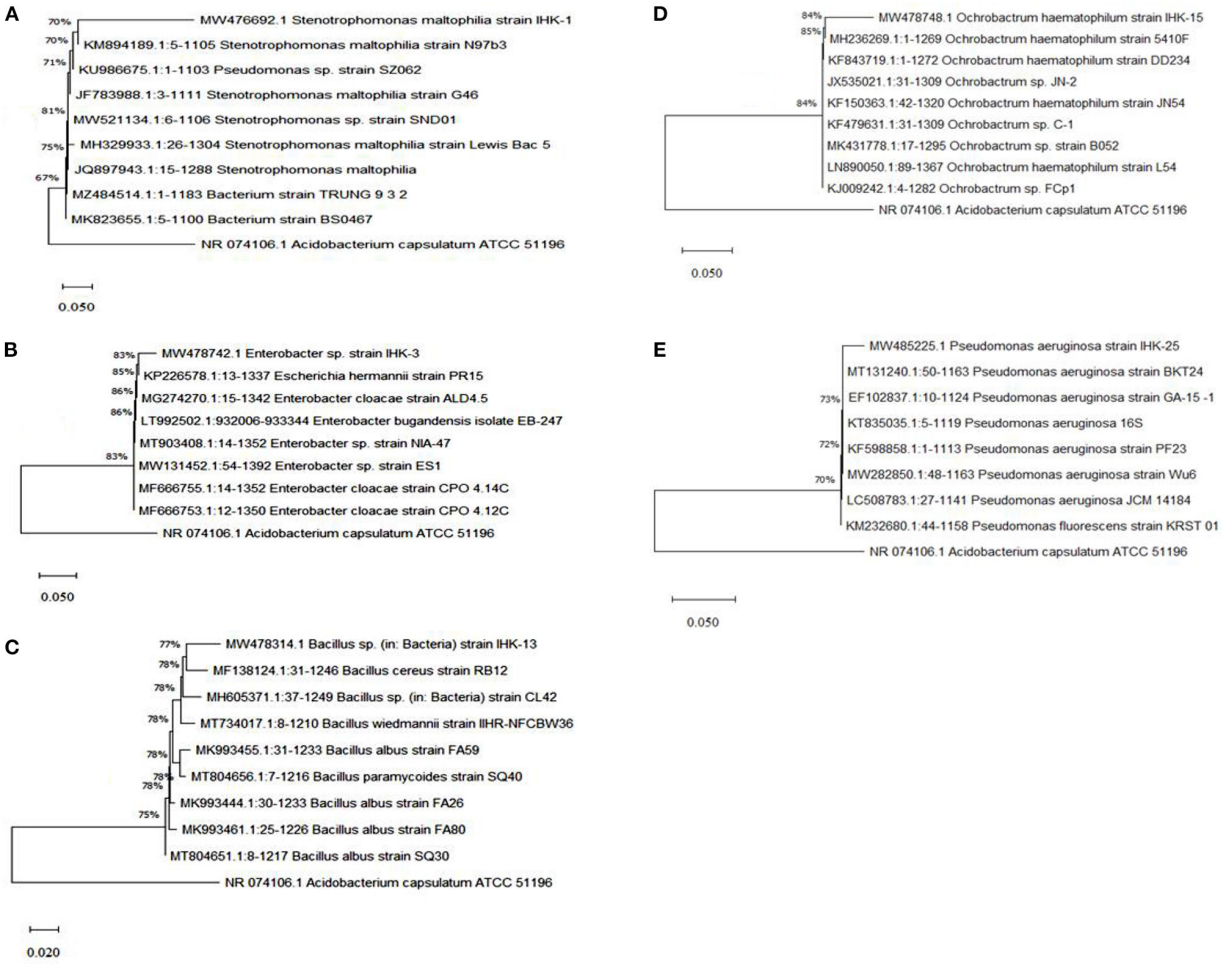
**(A–E)** Phylogenetic tree of the five isolates (IHK-1, IHK-3, IHK-13, IHK-15, and IHK-25) based on 16 S rRNA gene sequences. The neighbor-Joining method was used to construct the phylogenetic tree, and evolutionary analysis was conducted in MEGA X software and 16S sequence of *Acidobacterium capsulatum* (phylum Acidobacteria) was used as an outgroup. The bar represents 0.05 substitutions per site.

Out of fifteen bacterial isolates, five potential isolates labeled IHK-1, IHK-3, IHK-13, IHK-15, and IHK-25 displayed substantial PGP traits, antagonistic activity, lytic enzyme production and biofilm formation were chosen further for identification at the molecular level. The 16 S rRNA gene sequences obtained (1,303 bp for IHK-1, 1,349 bp for IHK-3, 1,232 bp for IHK-13, 1,296 bp for IHK-15 and 1,119 bp for IHK-25) were matched with homologous sequences from GenBank, aligned and the phylogenetic tree was constructed (Figure 2). The sequence of the isolate IHK-1 revealed 100% resemblance with *Stenotrophomonas maltophilia*, IHK-3 had 100% homology with *Enterobacter sp.*, IHK-13 showed 99% similarity with *Bacillus sp*., IHK-15 showed 99% sequence homology with *Ochrobactrum haematophilum* and IHK-25 showed 99% similarity with *Pseudomonas aeruginosa*. The nucleotide sequences of the bacterial strains were submitted to NCBI GenBank and accession numbers were received as follows: IHK-1: MW476692; IHK-3: MW478742; IHK-13: MW478314; IHK-15: MW478748; IHK-25: MW485225 (Supplementary Figure S7; Table 5).

**Table 5:** Identification of efficient diazotrophic PGP bacterial isolates by 16S rRNA gene sequencing analysis.

**Isolate label**	**Source of isolation**	**GenBank accession No**.	**Homologous microorganism**	**16S rRNA fragment length**	**% identity**
IHK-1	Rhizosphere	MW476692	*Stenotrophomonas maltophilia*	1303 bp	100%
IHK-3	Rhizosphere	MW478742	*Enterobacter* sp.	1349 bp	100%
IHK-13	Rhizosphere	MW478314	*Bacillus* sp.	1232 bp	99%
IHK-15	Rhizosphere	MW478748	*Ochrobactrum haematophilum*	1296 bp	99%
IHK-25	Rhizosphere	MW485225	*Pseudomonas aeruginosa*	1119 bp	99%

#### Plant Growth Promotion and Seed Germination of Rice by Selected Potential Diazotrophic Bacteria

**Figure 3:**
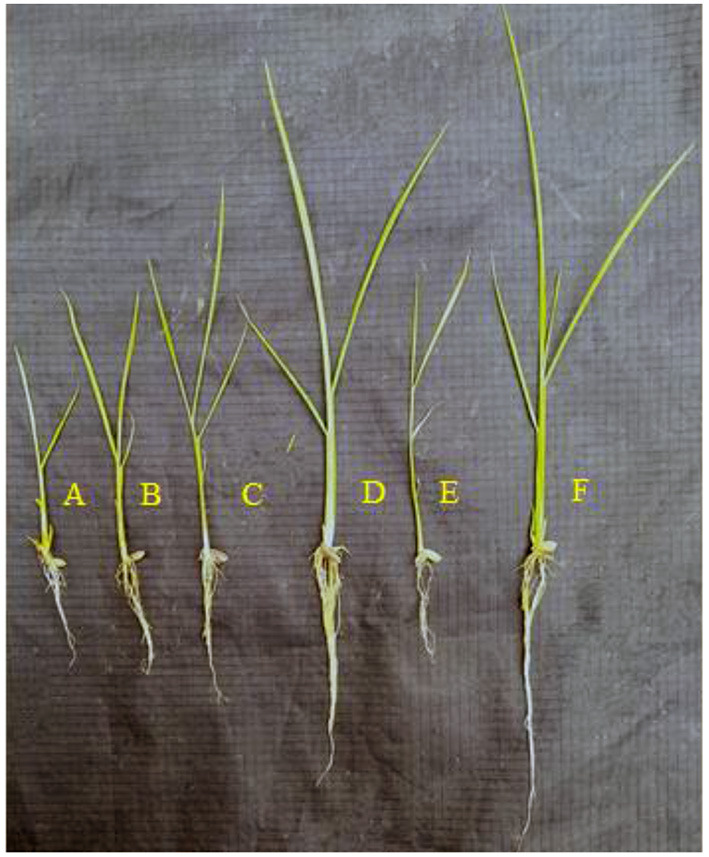
Growth promotion of rice treated with five selected diazotrophic PGP bacteria. **(A)** Negative control. **(B)** IHK-1. **(C)** IHK-3. **(D)** IHK-13. **(E)** IHK-15. **(F)** IHK-25.

Based on *in vitro* PGP traits, antifungal activity and biofilm formation, five potential isolates *Stenotrophomonas maltophilia* IHK-1*, Enterobacter* sp. IHK-3*, Bacillus* sp. IHK-13*, Ochrobactrum haematophilum* IHK-15 and *Pseudomonas aeruginosa* IHK-25 exhibited good results were selected to test on rice (BPT-5204) as host for seed germination and seedling vigor index utilizing the paper (roll) towel technique. Treatment of rice seeds with bacterial isolates individually showed significantly higher germination percentage, root and shoot growth, seed vigor index and total dry biomass of plants compared to control. The maximum seed vigor index was by *Pseudomonas aeruginosa* IHK-25 (2,264.3 ± 20), followed by *Bacillus* sp. IHK-13 (1,852 ± 50), *Stenotrophomonas maltophilia* IHK-1 (1,623.9 ± 44), *Ochrobactrum haematophilum* IHK-15 (1,521.7 ± 52) and *Enterobacter* sp., IHK-3 (1,399 ± 28). The percentage increase in seed germination was also in the same line. The maximum increase in seed germination was 36% by *Pseudomonas aeruginosa* IHK-25, followed by *Bacillus* sp. IHK-13 (32%), *Stenotrophomonas maltophilia* IHK-1 (28%), *Ochrobactrum haematophilum* IHK-15 (22%) and *Enterobacter* sp. IHK-3 (14%) compared to control. Similar results were also recorded with total dry weight. Treatment with *Pseudomonas aeruginosa* IHK-25 results in a maximum dry weight of 16.4 ± 0.3 mg, corresponding to increases of 59% followed by *Bacillus* sp. IHK-13 (50%), *Stenotrophomonas maltophilia* IHK-1 (49%), *Ochrobactrum haematophilum* IHK-15 (31%) and *Enterobacter* sp. IHK-3 (26%) compared to control (Figure 3; Table 6).

**Table 6:** Effect of potential PGP diazotrophic bacteria on rice seed germination and plant growth.

**Treatment**	**Root length (cm)**	**Shoot length (cm)**	**% Seed germination**	**Seed vigor index (SVI)**	**Plant dry weight (mg)**
Un- treated (Control)	6.1 ± 0.2^d^	6.4 ± 0.2^e^	72.7 ± 3.8^d^	908.7 ± 57^e^	10.3 ± 0.3^d^
*S.maltophilia* IHK-1	9.9 ± 0.1^b^	7.5 ± 0.2^cd^	93.3 ± 3.8^ab^	1623.9 ± 44^c^	15.3 ± 0.1^b^ (49%)
*Enterobacter* sp. IHK-3	8.3 ± 0.3^c^	8.5 ± 0.1^ab^	83.3 ± 1.9^c^	1399 ± 28^d^	13.0 ± 0.3^c^ (26%)
*Bacillus* sp. IHK-13	10.1 ± 0.1^b^	9.2 ± 0.3^a^	95.9 ± 1.2^ab^	1852 ± 50^b^	15.4 ± 0.1^b^ (50%)
*O. haematophilum* IHK15	9.7 ± 0.08^b^	7.4 ± 0.2^d^	88.9 ± 1.5^bc^	1521.7 ± 52^cd^	13.5 ± 0.3^c^ (31%)
*P. aeruginosa* IHK25	14.6 ± 0.1^a^	8.3 ± 0.2^bc^	98.8 ± 1.1^a^	2264.3 ± 20^a^	16.4 ± 0.3^a^ (59%)

*Numerical values are mean ± SE; mean values in each column with the same letter do not differ significantly at p < 0.05 and values in parenthesis are percent increase over control. Comparison between treatment mean values was made using Duncan's multiple range test (DMRT). Values in each column with the same letters do not differ significantly by DMRT (p = 0.05). Values in each column with different letters differ significantly by DMRT (p = 0.05)*.

## Discussion

The efficient PGP bacteria can be used as biofertilizers, biopesticides and phytostimulators to improve crop yield, soil quality and control phytopathogens. As a result, agricultural-based research mainly focuses on the rhizosphere, which has a diverse microbial community (22). In this study, a total of 15 bacteria were secluded from the rice rhizosphere on a combined carbon/ Rennie medium. Combined carbon medium provides the greatest populations of putative diazotrophic bacteria from rhizospheric soils (25, 26).

Out of fifteen bacterial isolates, five potential isolates labeled IHK-1, IHK-3, IHK-13, IHK-15 and IHK-25 displayed substantial PGP traits, antagonistic activity, lytic enzyme production and biofilm formation were identified as *Stenotrophomonas maltophilia, Enterobacter sp., Bacillus sp., Ochrobactrum haematophilum* and *Pseudomonas aeruginosa*. Earlier reports also include similar free living diazotrophic PGP bacteria from rice rhizosphere (7, 19, 23, 24, 47–49).

PGPR facilitate plant growth directly via solubilizing nutrients fixing atmospheric nitrogen producing phytohormones and indirectly by reducing the inhibitory impacts of different pathogens. Several studies have shown that using PGPR improved the health and production of various plant species under both normal and stressful situations (50, 51). Plant-beneficial rhizobacteria have the potential to reduce worldwide reliance on toxic agricultural chemicals that disrupt agro-ecosystems (11). In this study, 15 bacterial strains secluded from the rice rhizosphere were tested for multiple PGP traits under *in-vitro* conditions.

The N_2_ fixation ability of the isolated bacteria was evaluated by culturing the bacteria on NFB medium (nitrogen-free solid malate medium). The change in color of the medium is due to an elevation in pH caused by ammonia and nitrate production due to atmospheric N_2_ fixation. This preliminary method to test the nitrogen-fixing ability of bacterial isolates was also employed by Tan et al. (52), which were then confirmed by the ^15^N isotope dilution technique (53). The ability of the isolated bacteria to fix atmospheric nitrogen was further evaluated by the presence of *the nifH* gene in their genome. *nifH* gene codes for nitrogenase reductase, a vital component of the nitrogenase enzyme involved in nitrogen fixation. This enzyme is highly conserved, making it an effective molecular tool for determining biological nitrogen-fixing capacity in various environments. Out of 15 bacterial isolates, five isolates (IHK-1, IHK-3, IHK-13, IHK-15 and IHK-25) showed amplification with *the nifH* gene, and the product of predicted size (360-bp) was obtained, indicating the presence of nitrogen-fixing genes in these diazotrophic bacteria. The results are in accordance with the previous studies (54–60), applied Pol primers to detect the presence of *nifH* gene in various epiphytic and endophytic diazotrophic bacterial species such as *Bacillus, Pseudomonas, Enterobacter, Stenotrophomonas* and *Ochrobactrum* isolated from rice and other sources. The findings of nitrogen fixation by the ARA method indicated that a relatively large population of rice-associated nitrogen fixers are present in the soils and can improve the nitrogen nutrition of rice and other crops.

Many rhizobacteria are known to produce indole acetic acid extracellularly, which is a crucial phytohormone. The production has been associated with promoting plant growth, especially root elongation and initiation (61). For the synthesis of IAA, most plant growth-promoting bacteria require a precursor, tryptophan. Root exudates of many plants contain rich supplies of tryptophan, which PGPR utilizes for manufacturing and release auxins as secondary metabolites in the soil. In this study, out of 15 bacterial isolates tested, nine were able to produce IAA with the concentration ranging from 12.24 ± 2.86 to 250.3 ± 1.15 μg/ml (Table 2). These findings are higher than the previous reports of indole acetic acid production by PGP bacteria such as *Pseudomonas, Ochrobactrum, Enterobacter, Bacillus* and *Stenotrophomonas* isolated from the rice rhizosphere (1, 7, 19, 23, 24, 47, 48).

Phosphorus is one of the essential elements for plant growth and development. Since soil phosphorus is usually present in highly insoluble forms, it is regarded as a limiting nutrient for plant growth (62). With its capability to solubilize insoluble phosphate, PGPR has gained much interest in recent years because of its potential applications in agriculture to improve soil phosphorus bioavailability. Solubilization of phosphate is caused due to the secretion of microbial metabolites such as various organic acids, which lowers the pH of the culture media (22). In the present investigation, out of 15 bacterial isolates evaluated, six isolates showed phosphate solubilization with the concentration ranging from 36.69 ± 1.63 to 312.4 ± 1.15 μg/ml (Table 2). These findings are higher than the previous reports of phosphate solubilization by PGP bacteria such as *Bacillus, Ochrobactrum, Pseudomonas, Enterobacter* and *Stenotrophomonas* isolated from the rice, wheat and mung bean rhizosphere (1, 19, 23, 48, 63, 64). The pH of the medium also showed a decrease from 7.0 in most of the isolates. In bacterial isolates IHK-25, IHK-3 and IHK-15, the pH decreased from an initial 7.0 to a minimum of 3.5, 3.7, and 3.8, respectively (Table 2). This pH drop in the growth medium also differed among each isolate and could be related to the production of various organic acids by PSB. The decreased pH of culture filtrates was well-correlated with the increased level of orthophosphate in culture filtrate, as documented by several researchers (65).

Siderophores produced by PGPR have been demonstrated to promote plant growth by supplying iron ions to plants and limiting pathogen growth by putting pathogens under iron deprivation circumstances (66). In the present investigation, seven isolates produced siderophore, which is evident by the generation of orange halo zone on the CAS agar medium. In this study, all the 15 bacterial isolates were tested positive for ammonia production, and eight isolates showed HCN production (Table 2). HCN and ammonia production have been reported to play an essential role in the biological control of pathogenic fungi (22). Ethylene is a hormone produced in plants extensively under stressful environments like drought, salt stress, flooding stress, heavy metal stress and pathogen attack. Plant growth and development are adversely affected by higher levels of ethylene. Rhizobacteria showing ACC deaminase activity assist plants to endure stresses by lowering the concentration of ethylene. In the present investigation, six isolates (IHK-1, IHK-3, IHK-10, IHK-13, IHK-15 and IHK-25) showed ACC deaminase activity, demonstrating their ability to utilize ACC as a nitrogen source (Table 2). The same results were observed by various researchers (19, 24, 67, 68), who reported the ACC deaminase activity in various genera of *Pseudomonas, Ochrobactrum, Enterobacter* and *Bacillus*.

PGPR production of extracellular hydrolytic enzymes is a vital mechanism directed against phytopathogens for sustainable plant diseases management. The production of the wide range of hydrolytic enzymes by PGP bacteria in the soil also plays a vital role in enhancing the fertility of agricultural soils by hydrolyzing complex substances like polysaccharides, proteins, etc., into simpler forms added to soil again (22). In the present investigation, 73% of isolates showed protease activity, 60% amylase, 40% chitinase, 87% cellulase and 60% showed lipase activity. In addition, out of 15 bacterial isolates, 5 isolates (*Stenotrophomonas maltophilia* IHK-1, *Enterobacter sp.*, IHK-3, *Bacillus sp*., IHK-13, *Ochrobactrum haematophilum* IHK-15 and *Pseudomonas aeruginosa* IHK-25) were able to produce all the five enzymes (Table 3). The results of the present study correlate with the previous findings of Rasool et al. (22), who identified various PGP bacteria producing extracellular hydrolytic enzymes. Plants infected with various fungal diseases are a significant constraint in global agricultural production. To regulate phytopathogens, farmers usually use a variety of chemically synthesized inorganic fungicides. These inorganic fungicides are non-biodegradable and can remain in the soil for an extended period, results in soil infertility. Application of PGPR with substantial antifungal potency can be used as an alternative to achieve sustainable agriculture (69). The present bacterial isolates have been proven to be effective in limiting the mycelial growth of plant pathogens such as *R. solani* and *F.oxysporum* (Table 4). The same results were observed by various researchers (60, 64, 70–75), in several strains of *Pseudomonas aeruginosa, Bacillus* sp., and *Stenotrophomonas* sp., showed antagonistic effects against soil-borne fungi like *R. solani* and *F. oxysporum*.

Competition exists among microflora in the root region of plants to form biofilms on the root surface, which is a thick matrix of bacteria. In order to establish in the rhizosphere and boost plant development and yield, an efficient PGPR should bind to the root surface of plants by forming biofilms (76). There is evidence indicating that inoculation with biofilm-forming PGPR has better plant growth promotion than non-biofilm former inoculants (77). In this study, all of the bacterial isolates were tested for their potential to form biofilms under *in-vitro* conditions. Among 15 bacterial isolates studied, 5 (IHK-1, IHK-3, IHK-13, IHK-15 and IHK-25) formed very thick biofilms (Supplementary Figure S5). The results are in accordance with the previous studies carried out by Altaf and Ahmad (78), who reported that *Bacillus, Pseudomonas, Stenotrophomonas* and *Enterobacter* could form biofilms.

In the present investigation, five potential isolates IHK-1, IHK-3, IHK-13, IHK-15 and IHK-25, were chosen for testing on rice (BPT-5204) as a host for seed germination and seedling vigor index utilizing the paper (roll) towel method. The seeds treated with *Pseudomonas aeruginosa* IHK-25 showed significantly high germination percentage, root and shoot growth, seed vigor index and total dry weight, followed by *Bacillus* sp. IHK-13, *Stenotrophomonas maltophilia* IHK-1, *Ochrobactrum haematophilum, Enterobacter* sp., IHK-3 compared to non-inoculated seeds (Table 6). The results of this study were consistent with past findings that showed rice seeds treated with *P. aeruginosa, Bacillus* sp., *Ochrobactrum haematophilum, Stenotrophomonas maltophilia* and *Enterobacter* sp. significantly boosted rice seedling growth and yield (1, 7, 19, 23, 24, 48). Positive impacts of PGPR on rice plant growth may be due to the enhanced production of plant hormones like indole acetic acid, which might have stimulated the activity of enzymes like α-amylase that enhanced early germination by improving the levels of soluble sugars from starch breakdown (79), nitrogen fixation and P solubilization capability of the bacterial strain, and any others PGPR activities in favor of plant growth response.

## Conclusion

The present study concludes that five diazotrophic bacteria (*Stenotrophomonas maltophilia* strain IHK-1, *Enterobacter sp*. strain IHK-3, *Bacillus sp*. strain IHK-1, *Ochrobactrum haematophilum* strain IHK-15 and *Pseudomonas aeruginosa* strain IHK-25) isolated from rice rhizosphere had tremendous plant growth-promoting attributes, including N_2_- fixation, siderophore production, IAA production, inorganic phosphate, zinc, rock phosphate solubilization, ACC deaminase activity, ammonia production, HCN production, and lytic enzymes production. Furthermore, these bacteria have been demonstrated to exhibit substantial antagonistic activity against the phytopathogens *R. solani*, and *F. oxysporum* under *in vitro* conditions and have a positive effect on the growth of rice seedlings. Based on the findings of this study, it can be stated that rhizobacterial isolates such as IHK-1, IHK-3, IHK-13, IHK-15 and IHK-25 can be employed as bioinoculants in the cultivation of rice in a sustainable manner. In future studies these isolates can be subjected to field trials in order to improve the yield and available nutrients in the rice crop.

## Data Availability Statement

The datasets presented in this study can be found in online repositories. The names of the repository/repositories and accession number(s) can be found in the article/Supplementary Material.

## Author Contributions

MM: performed the experiments, analyzed and interpreted the data, and wrote the final version of the manuscript. BH: conceived and designed the experiments, analyzed the data, contributed reagents, and materials. HQ: wrote the draft. BK: conceived and designed the experiments and analyzed and interpreted the data. NI, AT, HE, DD, and AG: reviewed, revised and edited the manuscript. RS: conceived and designed the experiments, reviewed & edited the manuscript, analyzed the data, and wrote the final version of the manuscript, HK, HE, and AT: helped in fund acquisition. All authors contributed to the article and approved the submitted version.

## Funding

This work was funded by the Researchers Supporting Project Number (RSP- 2021/403), King Saud University, Riyadh, Saudi Arabia, RMC-UTM financial support from the industrial projects No. R.J.130000.7609.4C359 and R.J.130000.7609.4C240 and the Fundamental Research Grant Scheme (FRGS/1/2020/STG01/UPM/02/6 vote number 5540394) supported by the Malaysian Ministry of Higher Education, Malaysia.

## Conflict of Interest

The authors declare that the research was conducted in the absence of any commercial or financial relationships that could be construed as a potential conflict of interest.

## Publisher's Note

All claims expressed in this article are solely those of the authors and do not necessarily represent those of their affiliated organizations, or those of the publisher, the editors and the reviewers. Any product that may be evaluated in this article, or claim that may be made by its manufacturer, is not guaranteed or endorsed by the publisher.
