# Supplementary material for: Multifarious Indigenous Diazotrophic Rhizobacteria of Rice (Oryza sativa L.) Rhizosphere and Their Effect on Plant Growth Promotion
Source: Front Nutr. 2022 Jan 13;8:781764. doi: 10.3389/fnut.2021.781764 (PMC8793879; doi:10.3389/fnut.2021.781764)
Supplement: Supplementary file 1 [file Data_Sheet_1.docx]

**Supplementary Material**

**TABLE S1 |** Morphological characteristic of bacteria isolated from rhizosphere of rice

| **Isolates** | **Colony Form** | **Colony Margin** | **Colony Elevation** | **Colony Colour** | **Colony Texture** | **Optical density** | **Cell shape** | **Gram’s reaction** |
| --- | --- | --- | --- | --- | --- | --- | --- | --- |
| IHK-1 | Circular | Entire | Convex | Brownish | Smooth | Opaque | Rod shaped | -ve |
| IHK-2 | Circular | Entire | Convex | Brownish | Smooth | Opaque | Rod shaped | -ve |
| IHK-3 | Circular | Entire | Convex | Creamy | Smooth | Opaque | Rod shaped | -ve |
| IHK-4 | Circular | Entire | Convex | Creamy | Smooth | Opaque | Rod shaped | -ve |
| IHK-5 | Circular | Entire | Convex | Creamy | Smooth | Opaque | Rod shaped | -ve |
| IHK-6 | Circular | Entire | Convex | Creamy | Smooth | Opaque | Rod shaped | -ve |
| IHK-7 | Circular | Entire | Convex | Brownish | Smooth | Opaque | Rod shaped | -ve |
| IHK-8 | Circular | Undulate | Raised | Bluish green | Mucoid | Opaque | Rod shaped | -ve |
| IHK-9 | Circular | Entire | Raised | Creamy | Smooth | Opaque | Rod shaped | -ve |
| IHK-10 | Irregular | Entire | Raised | Milky white | Mucoid | Opaque | Rod shaped | +ve |
| IHK-11 | Circular | Undulate | Raised | Bluish green | Mucoid | Opaque | Rod shaped | -ve |
| IHK-12 | Circular | Entire | Raised | Creamy | Smooth | Opaque | Rod shaped | -ve |
| IHK-13 | Irregular | Entire | Raised | Milky white | Mucoid | Opaque | Rod shaped | +ve |
| IHK-15 | Circular | Entire | Raised | Creamy | Smooth | Opaque | Rod shaped | -ve |
| IHK-25 | Circular | Undulate | Raised | Bluish green | Mucoid | Opaque | Rod shaped | -ve |

**TABLE S2 |** Biochemical features of the isolated bacteria

| **Isolates** | **Cat** | **Oxid** | **Cit** | **NR** | **Gel** | **Urease** | **Ind** | **Mot** | **MR** | **VP** |
| --- | --- | --- | --- | --- | --- | --- | --- | --- | --- | --- |
| IHK-1 | + | + | + | + | + | - | - | + | + | - |
| IHK-2 | + | + | + | + | + | - | - | + | + | - |
| IHK-3 | + | - | + | + | - | - | - | + | - | + |
| IHK-4 | + | - | + | + | - | - | - | + | - | + |
| IHK-5 | + | - | + | + | - | - | - | + | - | + |
| IHK-6 | + | - | + | + | - | - | - | + | - | + |
| IHK-7 | + | + | + | + | + | - | - | + | + | - |
| IHK-8 | + | + | + | + | + | - | - | + | - | - |
| IHK-9 | **+** | **+** | **+** | **+** | **-** | **+** | **-** | **+** | **+** | **-** |
| IHK-10 | + | + | + | + | + | - | - | + | + | - |
| IHK-11 | + | + | + | + | + | - | - | + | - | - |
| IHK-12 | **+** | **+** | **+** | **+** | **-** | **+** | **-** | **+** | **+** | **-** |
| IHK-13 | + | + | + | + | + | - | - | + | + | - |
| IHK-15 | **+** | **+** | **+** | **+** | **-** | **+** | **-** | **+** | **+** | **-** |
| IHK-25 | + | + | + | + | + | - | - | + | - | - |

+, activity present; -, activity absent; Cat, catalase; Oxid, oxidase; Cit, citrate utilization; NR, nitrate reduction; Gel, gelatinase; Ind, indole; Mot, motility; MR, methyl red; VP, Voges Proskauer test.


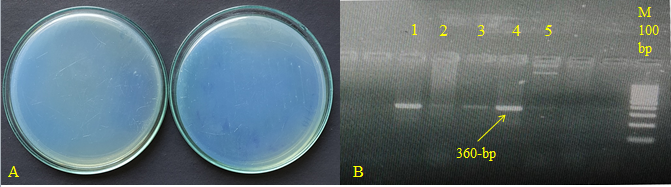


**FIGURE S1 |** Nitrogen fixing ability of isolated bacteria. (**A**) Blue colour of NFB media. **(B)** Amplification of *nifH* gene run on 1.5% agarose gel with an amplicon size of 360-bp. Lane 1, IHK-1; Lane 2, IHK-3; Lane 3, IHK-13; Lane 4, IHK-15 and Lane 5, IHK-25.


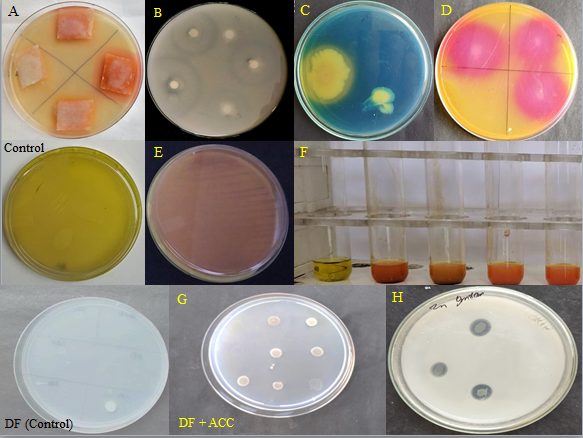


**FIGURE S2 |** PGP properties of diazotrophic bacteria isolated from the rhizosphere of rice. **(A)** IAA production by bacterial isolates evidenced by the pink color of nitrocellulose membrane. **(B)** Bacterial isolates showing halo zones on NBRIP media indicated solubilization of phosphate. **(C)** Orange halo zone on CAS agar plates indicates siderophore production. **(D)** Red coloration around the colonies indicated rock phosphate solubilization. **(E)** HCN production. **(F)** Ammonia production. **(G)** Isolates showing growth on DF medium amended with ACC indicating ACC deaminase activity **(H)** Bacterial isolates showing halo zones on tris mineral salt medium indicated zinc solubilization.


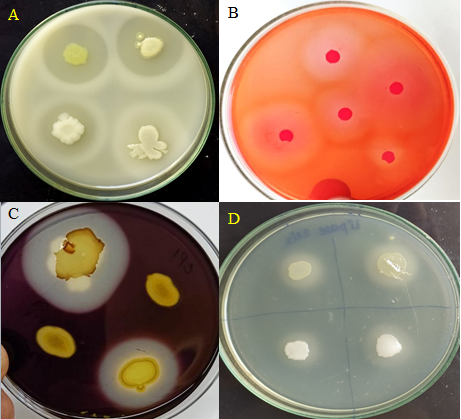


**FIGURE S3 |** Hydrolytic enzyme production by isolated bacteria. **(A)** Halo zone formation on skim milk agar medium indicated protease production. **(B)** Cellulase production. **(C)** Amylase production. **(D)** Lipase production.

**
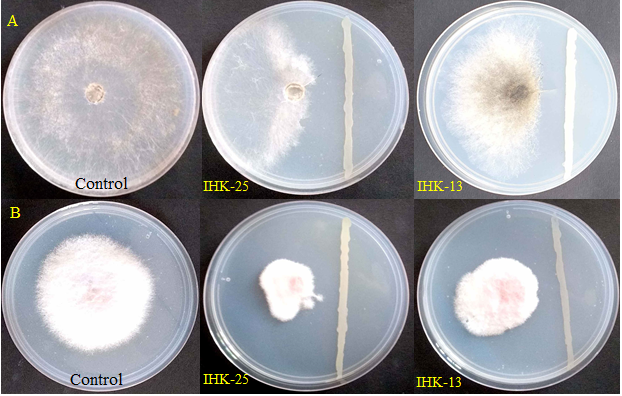
**

**FIGURE S4 | (A)** Antifungal activity of isolates against *Rhizoctonia solani*. **(B)** Antifungal activity of isolated bacteria against *Fusarium oxysporum*.


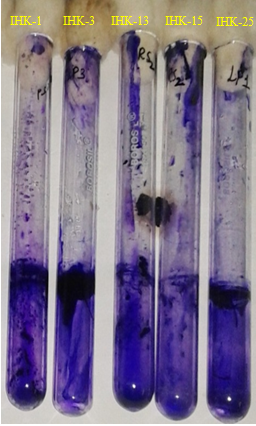


**FIGURE S5 |** Biofilm formation by diazotrophic PGP bacterial isolates


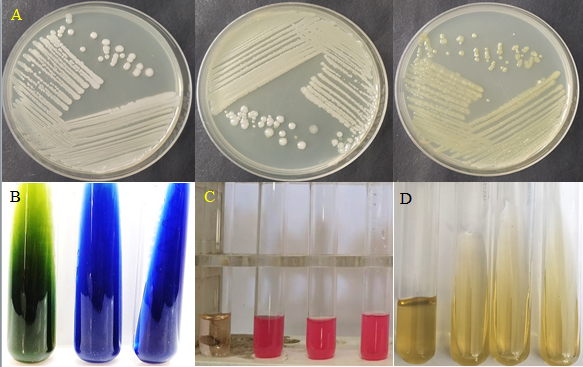


**FIGURE S6 | (A)** Few purified cultures of bacteria on nutrient agar medium. **(B)** Citrate utilization test. **(C)** Nitrate reduction test. **(D)** Gelatinase test.


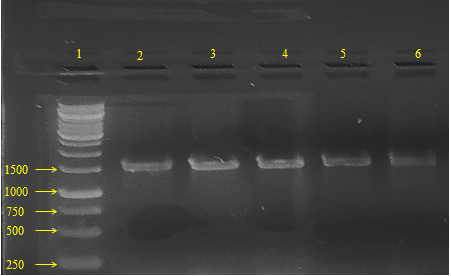


**FIGURE S7 |** 16S rDNA gene amplification with an amplicon size of 1500-bp. Lane 1, Ladder 1- kb ; Lane 2, IHK-1; Lane 3, IHK-3; Lane 4, IHK-13; Lane 5, IHK-15 and Lane 6, IHK-25.

| **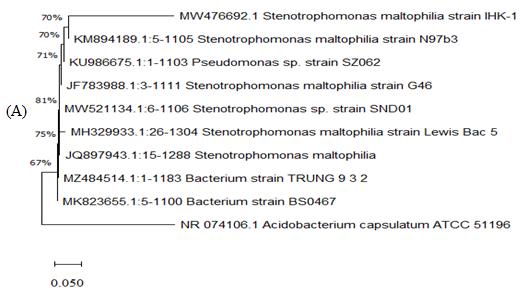** |
| --- |
| **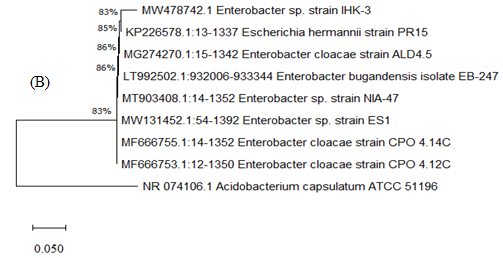** |
| **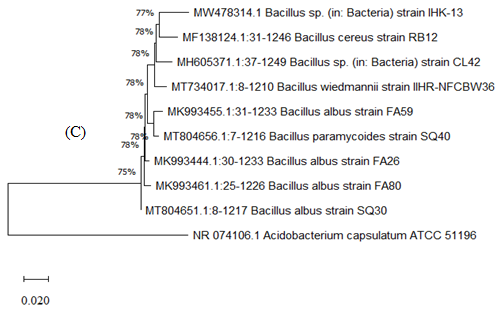** |
| **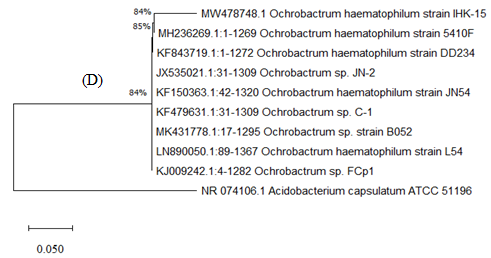** |
| **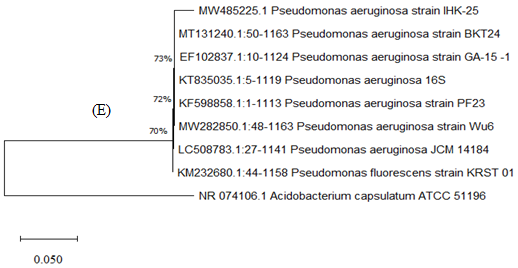** |

**FIGURE S8 |** Phylogenetic tree of the five isolates (IHK-1, IHK-3, IHK-13, IHK-15 and IHK-25) based on 16 S rRNA gene sequences. The neighbor-Joining method was used to construct the phylogenetic tree, and evolutionary analysis was conducted in MEGA X software and 16S sequence of *Acidobacterium capsulatum* (phylum Acidobacteria) was used as an outgroup. The bar represents 0.05 substitutions per site. The evolutionary distances were computed using the Maximum Composite Likelihood method and are in the units of the number of base substitutions per site.
